# Beyond building back better: imagining a future for human and planetary health

**DOI:** 10.1016/S2542-5196(21)00262-X

**Published:** 2021-11-10

**Authors:** Emilia Aragón de León, Amanda Shriwise, GÖran Tomson, Stephen Morton, Diogo Simão Lemos, Bettina Menne, Mark Dooris

**Affiliations:** a Health Policy Development and Implementation, World Health Organization Regional Office for Europe, Copenhagen, Denmark; b Health Emergencies Programme, World Health Organization Regional Office for Europe, Copenhagen, Denmark; c Political Economy of the Welfare State, Forschungszentrum Ungleichheit und Sozialpolitik, Research Centre on Inequality and Social Policy, Universität Bremen, Bremen, Germany; d Department of Sociology, University of Kansas, Lawrence, KS, USA; e President's Office, Karolinska Institutet, Stockholm, Sweden; f Swedish Institute for Global Health Transformation, Royal Swedish Academy of Sciences, Stockholm, Sweden; g Health and Sustainable Settings Unit, University of Central Lancashire, Preston, UK; h Institute of Citizenship, Society, and Change, University of Central Lancashire, Preston, UK; i Office for Investment for Health and Development, World Health Organization Regional Office for Europe, Venice, Italy

## Abstract

COVID-19 is disrupting and transforming the world. We argue that transformations catalysed by this pandemic should be used to improve human and planetary health and wellbeing. This paradigm shift requires decision makers and policy makers to go beyond building back better, by nesting the economic domain of sustainable development within social and environmental domains. Drawing on the engage, assess, align, accelerate, and account (E4As) approach to implementing the 2030 Agenda for Sustainable Development, we explore the implications of this kind of radical transformative change, focusing particularly on the role of the health sector. We conclude that a recovery and transition from the COVID-19 pandemic that delivers the future humanity wants and needs requires more than a technical understanding of the transformation at hand. It also requires commitment and courage from leaders and policy makers to challenge dominant constructs and to work towards a truly thriving, equitable, and sustainable future to create a world where economic development is not an end goal itself, but a means to secure the health and wellbeing of people and the planet.

## Introduction

COVID-19 is a defining global crisis, disrupting and transforming the world with profound consequences for governments, institutions, cities, communities, families, and individuals. Reflecting on the past 18 months, it is clear that COVID-19 and its containment measures have negatively affected the social, economic, and environmental domains of sustainable development and are threatening to reverse the progress on the 2030 Agenda for Sustainable Development and its 17 Sustainable Development Goals (SDGs).^1^ However, the pandemic has also revealed possible scenarios of a more sustainable world. As noted in a World Business Council for Sustainable Development report:^2^ “Like all crises, the COVID-19 pandemic has the potential to be a catalyst for positive change. Clearly, in the short term, its consequences are overwhelmingly negative…But precisely because it is so disruptive a shock to our economic and political systems, there is also the possibility that COVID-19 will help accelerate the emergence of…profound market shifts with exponentially positive consequences for people and planet.”

COVID-19 is a reminder that human health is inextricably connected to planetary, economic, and societal health and wellbeing. First, because this pandemic is believed to be zoonotic in origin,^3^, ^4^ it spotlights the human exploitation of nature, driven by an unsustainable food system linked to habitat destruction and biodiversity decline.^5^, ^6^ Second, it exposes the weaknesses in pandemic preparedness consequential to the interconnected global economy, travel, and trade,^7^ as well as gaps in social and health protection.^8^ Third, it reinforces that health crises can quickly become economic, social, humanitarian, and security threats.^9^, ^10^ Fourth, COVID-19 can be more accurately understood in most contexts as a syndemic, characterised by synergistic interaction between biological and social conditions and requiring action on wider determinants of health.^11^

At the same time, the disruption caused by COVID-19 has been transformative, showing how rapidly economic and social behaviours can change, providing glimpses of what a better world might look like and offering a window of opportunity to shape the future of sustainable development. Amid efforts to counteract the pandemic and prepare for recovery, at least three future scenarios are discernible. The first approach would be to simply build back by returning to traditional models of economic growth (eg, extraction, consumption, waste, and emissions).^12^ Although this approach might produce short-term benefits for some, it will constrain progress towards many SDGs and threaten humankind's collective future. The second approach, in line with the 2030 Agenda for Sustainable Development, would be to build back better by increasing the resilience of countries and communities and reiterating the need to balance the social, economic, and environmental domains of sustainable development.^13^ However, this incremental approach, even when green and inclusive,^14^ depends on the continued championing of current forms of economic growth and globalisation that are inadequate for preventing and addressing the root causes of pandemics, climate emergencies, and social injustices.^15^ The third approach would be to choose a radically transformative change^16^ that goes beyond building back better by advocating and generating a consensus for a nested model of sustainable development. Such a model embeds the economic domain within the social and environmental domains, in contrast to the dominant sustainability model of intersecting circles, which implies that all three domains are of similar and equal importance to sustainable development.^17^, ^18^ As opposed to more linear and incremental approaches, this scenario views the COVID-19 pandemic as an opportunity to transform or reconfigure the relationship between the three domains of sustainable development, instilling new norms that view the economy not as an end goal itself, but as a means to improve human and planetary health and wellbeing.^16^, ^19^ This approach thus calls for a regenerative and distributed model committed to social and ecological justice^20^ and appreciates that the “global economy services society, which lies within Earth's life-support system…on which the welfare of current and future generations depends”.^21^

**Key messages**

1 Although disruption caused by COVID-19 has negatively affected the social, economic, and environmental domains of sustainable development, it has also created new opportunities for building a more sustainable future.
2 In the context of growing concerns about climate change, biodiversity loss, and other challenges, these opportunities for transformative change should be urgently harnessed to improve human and planetary health.
3 Such change requires going beyond building back better by nesting the economic domain of sustainable development within social and environmental domains, thereby challenging conventional economic thinking by viewing economic development not as an end goal itself, but as a means to improve the health and wellbeing of people and the planet.
4 The engage, access, align, accelerate, and account (E4As) approach to implementing the 2030 Agenda for Sustainable Development provides a framework to explore how the COVID-19 pandemic could stimulate this reconfiguration and facilitate radical transformative change, highlighting the centrality of strategic engagement, leadership, and political commitment.
5 Recovery from the COVID-19 pandemic is about much more than the ability to contain the disease. It is symbolic of the commitment and courage to challenge the status quo, envision what it means to thrive as people and planet, and go beyond building back better to deliver the future that humankind wants and needs.

## Beyond building back better: using the E4As approach to progress human and planetary health

**Figure 1:**
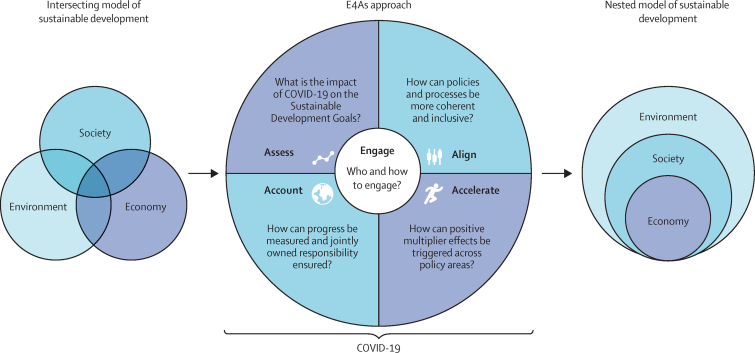
Transformative change from COVID-19 for human and planetary health E4As=engage, assess, align, accelerate, and account.

This Viewpoint builds on the third scenario, calling for a transformative change that goes beyond building back better. Through the use of the engage, assess, align, accelerate, and account (E4As) approach,^22^, ^23^ we explore and illustrate how COVID-19 disruption could enable a reconfiguration of the dominant model of sustainable development, facilitating progress towards the SDGs and promoting human and planetary health (figure 1). Developed in the WHO European region, the E4As approach was chosen as an appropriate exploratory framework because it is one of the first, and to our knowledge, the most recent policy framework to integrate societal transformative change with systems-level policy implementation at the intersection of health and sustainable development.^22^ Although our analysis of both COVID-19 effects and policy areas with a scope for alignment was focused on the WHO European region, our aim has been to take the whole planet into consideration, and so this Viewpoint also draws on relevant global evidence. We also recognise that European actions have effects on a global scale and on planetary boundaries (eg, climate change, biodiversity loss, and air pollution), and that global decision making influences European policy and action.

To imagine how COVID-19 disruption could catalyse transformative change and take us beyond building back better, we used five key concepts. The first concept is assessment, which focuses on evaluating the progress towards achieving the SDGs, and understanding the effects of COVID-19 and opportunities in relation to these. The second concept is alignment, which concerns harmonising policies and processes related to the achievement of the SDGs, both within and between sectors and levels of governance, and considers how COVID-19 disruption could enable a coherent, normative shift towards a nested model of sustainable development. The third concept is acceleration, which considers how positive multiplier effects can be triggered across policy areas to enhance progress towards the SDGs, illustrated with references to three examples (wellbeing economies, social movements, and digital technological innovations) spotlighted by the pandemic. The fourth concept is accountability, which highlights the need for policy making to embed new metrics that can track progress towards human and planetary health, recognises that achieving the SDGs can be a jointly owned responsibility across sectors and levels, and emphasises that recovery and transition from the COVID-19 pandemic can likewise harness the tangible commitment of multiple actors. The fifth concept is engagement, which refers to the meaningful and systematic involvement of relevant stakeholders across all sectors and levels in the planning, conduct, dissemination, uptake, and evaluation of policies and interventions for human and planetary health, appreciating that sustained dialogue and participation is a prerequisite for the transformative change that enables going beyond building back better.

We sought to empirically ground this semi-structured exploration. First, guided by the policy responses in the WHO European region,^24^ we conducted a rapid narrative review of academic and grey literature published between December, 2019, and October, 2020, that addressed COVID-19, human and planetary health, health governance, and the build back better policy discourse. We categorised the potential effects of COVID-19 on the SDGs according to the three domains of sustainable development: social (SDGs 1–5), economic (SDGs 7–12), and environmental (SDGs 6 and 13–15); and also considered the effects on institutions (SDG 16) and partnerships (SDG 17).^25^, ^26^ Second, we identified three illustrative functional policy areas (food systems, transport and mobility, and work and incomes) to explore these effects in greater depth. These policy areas were chosen as illustrative examples because they had extensive disruption early on in the pandemic because of containment measures and were deemed to have a high amount of political importance because of their effect across all three domains of sustainable development. Third, building on findings from the rapid narrative review and an in-depth examination of these illustrative functional policy areas, we identified accelerators with the potential to trigger multiplier effects and facilitate a shift towards a nested model of sustainable development. Fourth, we interpreted these findings in relation to the E4As approach, focusing on what is distinctive about going beyond building back better and on what will be required to shape recovery and transition planning to secure transformative change that aligns with the nested model.^17^, ^18^ For further information on the rapid narrative review, search strategy and selection criteria, justification for our categorisation of the SDGs, and a justification for our selection of the illustrative functional policy areas and accelerators, see the appendix (pp 1–4).

## Assess

**Figure 2:**
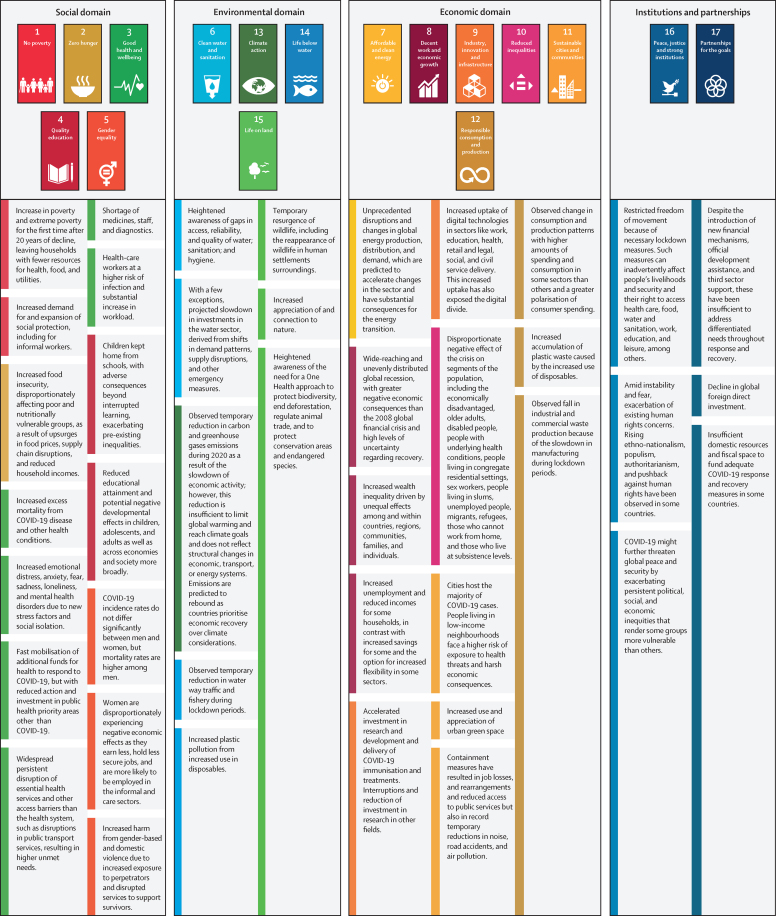
COVID-19 effects on health and sustainable development See the appendix (pp 5–7) for the references corresponding to these statements.

Before the COVID-19 pandemic, projections indicated that no WHO European region country was on track to achieve the health-related SDGs and targets and that implementation needed to be strengthened and better coordinated to accelerate progress.^27^, ^28^ Findings from our rapid narrative review (appendix pp 5–7) suggest that although catalysing some short-term positive changes and stimulating discourse about the potential for reimagining and reconfiguring the future,^20^ COVID-19 has largely affected the SDGs negatively, spotlighting and exacerbating pre-existing inequalities and threatening development gains (figure 2).^29^

In the social domain, COVID-19 has caused substantial excess morbidity and mortality in many countries,^30^ and vaccination programmes, sexual and reproductive health services, and chronic disease management have been severely disrupted.^31^ The pandemic has also resulted in increased food insecurity, disproportionately affecting poor and nutritionally vulnerable groups.^32^ Children and students have been learning from home because schools and universities across Europe are closed, with adverse consequences beyond the immediate educational effects.^33^ Although mortality rates are higher for men, women are more likely to bear the brunt of the pandemic's severe social and economic consequences.^34^ Alongside these negative effects, countries showed how quickly health and social protection benefits can be universalised and made more comprehensive.^8^, ^35^ Some population groups have also seen improvements in wellbeing linked to an increased appreciation of and connection to nature^36^ and the option for increased flexibility through remote working.^37^

In the environmental domain, the reduced industrial and commercial demand for fresh water and a decrease in anthropogenic greenhouse gas emissions (estimated at 6% for 2020)^38^ were observed after stringent lockdowns and the almost total cessation of production in many countries.^39^ However, such positive effects were short-lived. Countries, cities, and communities have faced difficulties in properly managing waste from the pandemic (eg, masks, gloves, and food packaging), resulting in its accumulation on beaches and in rivers and oceans.^40^, ^41^ Negative longer term consequences are forecast, with pressure for economic recovery encouraging the accelerated exploitation of the planet for shorter term gains.^41^

**Figure 3:**
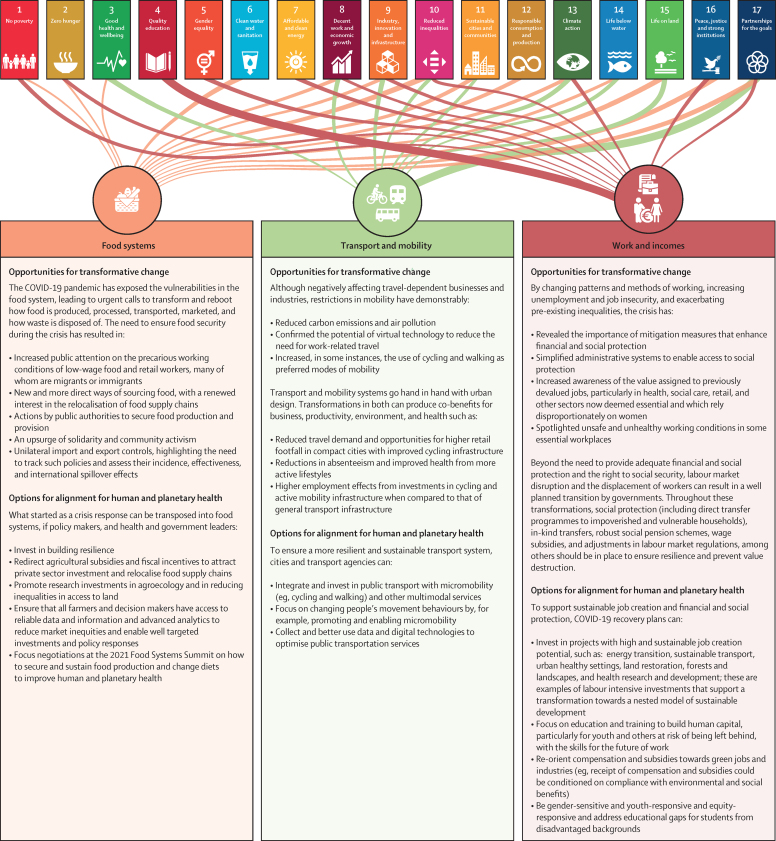
Illustrative examples of functional policy areas that have been disrupted by COVID-19, with the scope for alignment during recovery and transition See the appendix (pp 8–9) for the references corresponding to these statements.

In the economic domain, the pandemic threatens progress towards equitable prosperity because the burden caused by COVID-19 and its containment measures are distributed unequally, more severely affecting vulnerable and marginalised groups and thus amplifying inequalities.^29^ COVID-19 has severely affected existing infrastructures and services, limited mobility, and exacerbated unemployment and decreased productivity, with losses in working hours as high as the equivalent of 55 million full-time jobs in Europe and central Asia in the second quarter of 2020.^8^, ^42^ Economic contractions might limit the fiscal space and decrease the confidence required for the prioritisation of long-term investments in and structural adjustments for human and planetary health. Alongside many negative effects, particularly for vulnerable groups, the disruption has influenced production patterns^40^ and consumer behaviour,^43^ and increased investments in research and development and the uptake of innovation and technology, showing what is possible for the future of health care and environmental innovation.^44^, ^45^ These disruptions also present opportunities to change how economies function and to shape COVID-19 recovery for the wellbeing of people and the planet; for example, through the illustrative functional policy areas in figure 3 (appendix pp 8–9).

## Align

Because countries use multiple policy actions to mitigate the negative effects of COVID-19, it cannot be assumed that an alignment of these policies towards human and planetary health will happen naturally, or even that it is a likely outcome of recovery and transition processes. Despite widespread recognition that health policy challenges are intersectoral, requiring crosscutting and integrated approaches, an analysis of the Voluntary National Reviews of the countries from the WHO European region revealed the minimal use of such approaches or of legal and regulatory frameworks to progress the SDGs.^46^

Current narratives on building back better draw heavily from pre-COVID-19 UN resolutions; and, reflecting the traditional intersecting model of sustainable development, include discussions of mainstreaming^47^ and balancing^48^ the three domains. We contend that achieving the SDGs requires a commitment to going beyond this model through an alignment process that challenges the dominant conceptualisation of sustainable development by consciously nesting the economic within the social domain, which in turn sits within the life-supporting environmental domain. This framing reorients the recovery focus away from returning to business as usual,^49^ and towards reconfiguring the economy to better support the health and wellbeing of people and the planet. Robins^50^ reflected that, globally, there were “$379 trillion trillion dollars—more than enough to deliver a rapid transition to a resilient, just and zero emissions economy by the middle of this century”, but only if the incentives are changed that currently make it more profitable “to bet against the planet and ignore human development.” Normative assumptions about economic growth should be challenged alongside policy coordination and coherence across sectors and levels of governance, particularly with respect to fiscal strategy, regulatory responses, investments, and value creation.^51^, ^52^ Governments and policy makers ought to invest in building capacities and capabilities from within, through transparent interactions with other value creators in society to design new social contracts based on the ideas of public value and long-term resilience, as argued by Mazzucato and colleagues.^53^

The examination of a few illustrative functional policy areas that, in our assessment, have been extensively disrupted by COVID-19 shows the potential to facilitate meaningful and synergistic progress across the SDGs and towards a nested model that promotes human and planetary health (figure 3; appendix pp 8–9). For example, transport affects all domains of sustainable development and several health-related goals.^54^ A strategically aligned approach would consider how to create positive incentives for modal shifts at the same time as raising revenues, creating jobs, directing innovation and investment towards more balanced and integrated transport systems, and achieving the full potential benefits of active travel. This approach might include: removing fossil fuel subsidies, introducing tax-exempt transit benefits, and prioritising location-efficient development and investment in innovative and high-quality green public transport and urban infrastructure, at the same time as resisting a harmful return to the unsustainable mass use of cars and aeroplanes.^55^ This change also highlights intergenerational policy coherence: decisions made during the next decade will influence generations to come, and there are clear opportunities to connect policies on travel to those on climate change, green economies, equity, health and wellbeing, habitat protection, and biodiversity beyond 2030.^56^

## Accelerate

The complexity of the inter-relationships between SDGs requires systemic and transformative multipliers that reorient the economy towards human and planetary health.^57^, ^58^ To make the most of the opportunity offered by COVID-19 to go beyond building back better, we highlight three illustrative accelerators (wellbeing economies, social movements, and digital technological innovations) that offer the potential to progress a nested model of sustainable development and activate change across multiple policy areas, including those in figure 3 (appendix pp 3–4). Although momentum in all three of these accelerators was present before the pandemic, COVID-19 has spotlighted their potential to trigger positive multiplier effects towards (or away) from a nested model.

The recognision that the dominant economic models “aggravate the climate and ecological crises, and they perpetuate vastly unequal distributions of power and wealth”, as noted by Büchs and colleagues,^52^ advocates for wellbeing economies, which are shown by Raworth's Doughnut Economics model,^59^ which embraces the nested vision of sustainable development advocated for in this paper. Drawing on the SDGs, this model combines planetary boundaries (eg, climate change, biodiversity loss, and air pollution) and social boundaries (eg, health, food, and work), portraying sustainability as being ecologically safe and socially just, because resource use enables human thriving within environmental limits. In envisioning new paths forward, Raworth's model challenges the mantra of growth for growth's sake, and proposes a shift from linear economies that take, make, use, and lose, to circular economies that restore, regenerate, and reconnect humanity with the biosphere.^59^ Fanning and colleagues^17^ and O’Neill and colleagues^18^ illustrate how this shift in thinking and the adoption of wellbeing economies can activate and thereby accelerate action across policy areas such as transport, food, work, and health, and can be pursued through enhancing resource sufficiency to meet human needs and reduce overconsumption, where appropriate using degrowth and steady-state economy models;^60^, ^61^ and improving interacting systems for physical (eg, decarbonising energy and transportation and increasing crop-based diets) and social (eg, prioritising income equity, and pursuing universal health coverage and social protection) provisioning.

Public engagement and social movements, which have been pivotal in challenging traditional models of sustainable development and the mantra of economic growth,^62^ provide a second means to accelerate beyond building back better. The pandemic has prompted societal action to complement and catalyse governmental action,^63^ demanding better protection for essential workers, shifts in employer–employee relationships, and a healthy and green recovery strategy from COVID-19.^64^, ^65^ These demands are exemplified in a WHO manifesto,^66^ highlighting policy areas in need of radical change, such as food and transport, and calling for a global movement for health and the environment, as well as in a letter sent to G20 leaders from more than 350 organisations representing half of all medical professionals worldwide.^67^ Activist movements, such as Extinction Rebellion^68^ and School Strikes^69^ for example, have produced the widespread mobilisation of people demanding a healthy and just future on a liveable planet, calling for action on a range of policy areas (including those elaborated on in figure 3). To convert this momentum into substantive policy change for human and planetary health, these social movements aim to show the redundancy of traditional models of sustainable development and to advocate for their reconfiguration across all sectors and levels of governance.

Acceleration can also be triggered through digital technological innovation, applied in multiple interconnected policy areas. Artificial intelligence, the internet of things, big data, and blockchain technology can help to avert a future pandemic by supporting epidemic prevention and control, increasing the efficiency, security, and transparency of outbreak reporting systems, and reducing the spread of health misinformation, among other benefits in public health, health research, and medical practice.^44^, ^45^ More widely, the COVID-19 pandemic has highlighted the transformative role of digital innovation within education and work arenas. Digital technologies also have substantial potential for application in food, transport, and urban development systems: their use has been linked to more sustainable and effective agri-food systems, increased urban food security, improved logistics, greener transport solutions, and smart healthy cities.^70^, ^71^, ^72^, ^73^ Although new technologies can accelerate progress towards better human and planetary health, they should be based on models and values that account for structural biases, anticipate risks, and distribute benefits fairly across society. So as not to reinforce and magnify socioeconomic vulnerabilities and inequities, technological developments should account for the growing digital divide: it is crucial to tackle the widening gap between those who can access and profit from technological innovations and those who cannot. For example, older and younger people, those with a disability, and minority ethnic communities might be at particular risk of not being able to benefit from technological innovations.^74^ Orienting present and future technology access and use to move beyond building back better thus requires anticipatory and regulated policy making committed to closing the digital divide and to combating inequities within and across generations.^75^, ^76^

## Account

The non-binding and complex nature of the 2030 Agenda requires strong commitment and accountability at the global, national, and subnational levels, as well as platforms or mechanisms that measure progress, increase transparency, address power asymmetries, and make institutions more responsive.^77^ In addition to strengthening and using existing processes (eg, national, regional, and global accountability mechanisms, human rights instruments, and sanctions) in line with SDGs 16 (peace, justice, and strong institutions) and 17 (partnerships for the goals), *The Lancet*–University of Oslo Commission on Global Governance for Health^78^ has proposed a UN Multistakeholder Platform on Global Governance for Health. Such a platform would enable action for human and planetary health and address persistent inequities and weaknesses in global institutions that, if left unaddressed, might reinforce tendencies to simply build back. The UN Regional Coordination Mechanism for Europe and Central Asia's Issue-Based Coalitions focused on thematic areas such as health, social protection, gender equality, youth and adolescents, environment and climate change, sustainable food systems, the large movements of people, displacement, and resilience that could be strengthened to enable a radically transformative COVID-19 pandemic recovery, promoting appropriate reforms as necessary.^79^ Furthermore, intergovernmental coordinating mechanisms for environmental policy, such as the Intergovernmental Panel on Climate Change, established in 1988, and the Intergovernmental Science-Policy Platform on Biodiversity and Ecosystem Services, established in 2012, have provided platforms through which to advance environmental policy at increasingly high levels.

Nationally, all WHO European region countries have multisectoral coordination and accountability mechanisms in place to support the implementation of the 2030 Agenda.^27^ After the pandemic, these mechanisms could be used to prevent a siloed recovery driven predominantly by economic concerns, promoting instead a more transparent, participatory, and multisectoral transition that strengthens and institutionalises the reorientation of economic actors to social objectives and a respect for planetary limits.

Accountability mechanisms should also address inequities and leave no one behind. Individuals in vulnerable groups often face risks spanning multiple policy areas and sustainable development domains (figures 2, 3). A start to strengthening accountabilty is to actually measure what matters. COVID-19 pandemic recovery and transition plans can consider how these groups might be negatively affected and should systematically adopt equity-sensitive and equity-responsive policies and mitigation measures.^80^ Measuring progress towards human and planetary health that goes beyond building back better not only requires comparable and disaggregated data, but also demands the reassessment and revision of concepts underlying existing measures of progress. As Costanza and colleagues^81^ reflect, critiques of and calls to change gross domestic product as a signpost of economic and societal performance have proliferated since the 2008 crisis. In highlighting the disadvantages of its use to measure either human progress or social wellbeing, alternatives proposed include adjusted economic measures, subjective wellbeing measures, and weighted composite measures.^81^ Exploring the pathways between sustainable development and human wellbeing, de Neve and Sachs^82^ highlight income, social support, generosity, freedom, trust in government, and health as key determinants, and also advocate for research and policy related to both the SDGs and subjective wellbeing to be combined to accelerate sustainable development and ensure an integrated focus on people and planet.

New measures of progress have already been proposed. The Happy Planet Index (which is calculated using life expectancy × life satisfaction × equity factor) has been proposed as one way forward,^83^ measuring sustainable wellbeing for all with a focus on human and planetary health per unit of ecological footprint.^84^ The Organisation for Economic Co-operation and Development's Better Life Index has also been proposed, based on 11 topics identified as essential in the areas of material living conditions and quality of life.^85^ As shown by leadership in countries such as Iceland, New Zealand, and Scotland, who launched the Wellbeing Economy Governments Alliance at the 2018 Organisation for Economic Co-operation and Development's World Forum,^86^ health stakeholders have a role to play in measuring what matters and advocating for corresponding shifts in conceptualising and measuring wellbeing, progress, and economic and social performance.

## Engage

**Figure 4:**
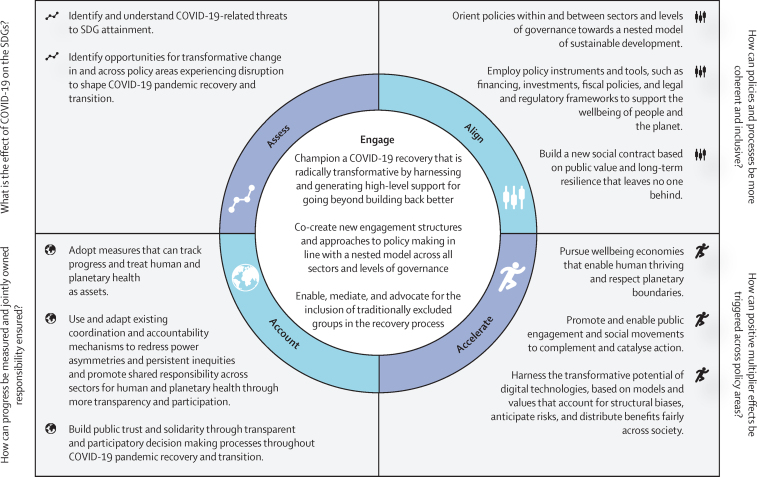
Beyond building back better through the E4As approach E4As=engage, assess, align, accelerate, and account. SDG=sustainable development goals.

Engagement is required throughout the four As (assess, align, accelerate, and account), which serve as entry points for transformative change that goes beyond building back better. The COVID-19 pandemic has disrupted the ideational and institutional rigidity that often constrains engagement. This disruption offers the potential to reform and generate new structures and approaches to policy making that nest the economic within the social and environmental domains and accelerate progress for human and planetary wellbeing (figure 4).

Across all sectors and levels, the disruption caused by the COVID-19 pandemic has also created a window of opportunity for transformation in the actor constellations (the group of key actors and relationships between them) shaping recovery and transition, with examples of previously peripheral actors taking up central roles in policy and governance (appendix pp 8–9). For example, key actors from the health sector now have a strong and central voice in recovery planning after being sidestepped in relation to the financial crisis after 2008, and population displacement crises of 2015 and after, which have had substantive effects on health across Europe and other regions.^87^ Given its new and indisputably central role in responding to the pandemic, the health sector should use its influence to engage in and champion COVID-19 pandemic recovery and transition that is radically transformative. This goal “requires changes in social structures and relations, including addressing the growing economic and political power of elites and patterns of stratification related to class, gender, ethnicity, religion or location that can lock people (including future generations) into disadvantage and constrain their choices and agency” as well as “changing norms and institutions, both formal and informal, that shape the behaviour of people and organizations in the social, economic, environmental and political spheres”.^16^ Together, these quotes imply a focus on mechanisms that are themselves transformative, advocating whole-of-government and whole-of-society governance mechanisms^88^ and exploring the effective use of participatory and deliberative democratic processes.^89^

Many questions related to transformative engagement for human and planetary health should be urgently addressed to ensure that there is a move to beyond building back better. For example, what role will the health sector have in allocating financial and other resources to support COVID-19 pandemic recovery and transition? How will the health sector engage in conversations with the International Monetary Fund, World Bank, other financial institutions, parliaments, and private sector bodies to transform the notion of building back better into a process that delivers a more healthy, just, equitable, and sustainable future? Will the health sector be brought into direct conversation with governments, employers, and education systems to help children whose learning suffered during the pandemic, support families who have lost breadwinners, and ensure the safety of workers and students as countries attempt to reopen and recover without exacerbating health inequities? Will the health sector leverage its potential and renewed position in central decision making to advocate for a proper environmental and social focus throughout the recovery and transition, including by engaging vulnerable and marginalised groups in the decision making process? Will these engagements be done in a way that builds public trust and solidarity as well as a new social contract for human and planetary health? The nature of these political engagements and their outcomes have consequences not only for recovery, but also for ensuring that no one is left behind and for institutionalising the development framework that emerges from the crisis.

65% of the SDG targets will not be achieved unless there is effective coordination with subnational governments, highlighting the crucial role of local authorities in establishing and delivering social and environmental objectives.^90^ The health sector should enable, mediate, and advocate for the promotion of health and wellbeing and ensure that communities, particularly those that have historically been excluded, have a voice and are informed and engaged in the recovery process, and benefit from mitigation measures proportionate to need. To engage effectively and strengthen long-term accountability, health stakeholders should use the disruption caused by the COVID-19 pandemic as a window of opportunity to facilitate action by strengthening community engagement for health and public health and emergency preparedness and response capacities in local agencies. A continuity in political commitment to the SDGs, steered from the highest level of government and supported by subnational and local governmental and societal actors, is crucial to support participation and to inform future multilevel approaches to governance and policy implementation.^91^, ^92^

## Conclusion

In this Viewpoint, we have argued that the COVID-19 pandemic offers a transformative opportunity to hasten progress for human and planetary health. However, this potential will only be realised if calls to simply build back are rejected and calls to build back better are recognised to be, for the most part, a green-tinged version of the same calls, rooted in the outdated and unsustainable belief that economic growth alone equates to progress. We have therefore focused on going beyond building back better, contending that this approach of necessity nests the economic within the social and environmental domains of sustainable development, viewing the economy not as an end goal itself but as a means to secure and improve the health and wellbeing of people and the planet.^17^, ^18^

Evidence and support for a pursuit of a nested model of sustainable development has only been furthered by the COVID-19 pandemic. The effect of the pandemic has increased awareness of the vulnerability of human health to zoonoses, at a time when there is increasing evidence of the imminent risks to human and planetary health and wellbeing from ecological threats such as climate change and biodiversity loss. It is, therefore, crucial that research and development activities on the prevention of future catastrophes are accelerated, as well as those on timely and effective responses. This change also requires continued academic engagement on the nature of transformative change and the changes required to go beyond building back better. For example, Scoones and colleagues^93^ have illustrated how “different ways of understanding what we mean by transformations can affect what actions follow”. Furthermore, social scientists have long recognised that transformations are not inherently good, and hence, they require “deliberate normative steering”.^93^ In other words, how recovery and transition from the COVID-19 pandemic are approached and considered matters, not least because a transformation towards a nested model of sustainable development will not naturally happen of its own accord.

Moving forward, it is therefore crucial to ensure that opportunities to reconfigure the relationship between the three sustainable development domains are not missed,^94^ obscured by all-consuming reactive efforts to address immediate economic needs. Even during more normal conditions for policy making, politically strategic approaches to health policy analysis and implementation tend to be neglected in favour of a search for technical solutions.^95^ In the same way that policies will not naturally align towards improving human and planetary health, the severity of the crises resulting from COVID-19 will neither naturally build a consensus on a vision for a better future, nor incentivise engagements to build it. Furthermore, Scoones and colleagues^93^ recognise that addressing immediate economic needs and moving towards a nested model of sustainable development need not be mutually exclusive and can instead be “complementary and reinforcing”.^93^ Moreover, the work of Meadows^96^ on leverage points to intervene in a system might have a renewed relevance in triggering multiplier effects and devising specific ways to activate transformations towards a nested model of sustainable development.

Strategic engagement, leadership, and political commitment are required to deliver on this shared vision of the post-COVID-19 world. To maximise its potential role in moving beyond building back better, the health sector should view engagements related to COVID-19 recovery and transition as opportunities to assess the effects and opportunities arising from the crisis; align policies across sectors towards a nested model of sustainable development; promote interventions to accelerate progress towards human and planetary health; challenge and reform pre-existing institutional coordination and accountability mechanisms at all levels of governance to prioritise equity, participation, and transparency; and to normalise new measures of progress that embrace holistic wellbeing.

Reflecting findings of *The Lancet–*University of Oslo Commission on Global Governance for Health,^78^ commissions and advisory bodies such as the Pan-European Commission on Health and Sustainable Development,^97^ the Independent Panel for Pandemic Preparedness and Response,^3^ *The Lancet* COVID-19 Commission,^98^ and the Council on the Economics of Health for All^99^ can play key roles, first as knowledge brokers, communicating independent and transparent multidisciplinary evidence to the UN and other actors for global governance for health; and, second, in activating transformations in financial and technical support for human and planetary health. These forums provide crucial support to policy makers to advocate, develop, and implement radically transformative policy agendas to be applied in global, regional, national, and local contexts. Recovery and transition beyond the COVID-19 pandemic are thus inextricably linked not only to investment and resource mobilisation for health and sustainable development, but also to inequality and the contentious nature of redistributive politics. From this viewpoint, COVID-19 pandemic recovery is about much more than the ability to contain and control the disease; it is symbolic of the commitment and courage to challenge the status quo, envision what it means to thrive as people and planet, and go beyond building back better to deliver the future that is wanted and needed.

## Declaration of interests

EAdL reports grants from Bundesgesundheitsministeriums fur Gesundheit, Germany, outside the submitted work. AS reports grants from the European Research Council, during the conduct of the study; personal fees from the Sustainable Development and Health Programme, WHO Regional Office for Europe, and the Office for Investment for Health and Development, WHO Regional Office for Europe, outside the submitted work. All other authors declare no competing interests.
