# Supplementary material for: Beyond building back better: imagining a future for human and planetary health
Source: Lancet Planet Health. 2021 Nov 10;5(11):e827–39. doi: 10.1016/S2542-5196(21)00262-X (PMC8600369; doi:10.1016/S2542-5196(21)00262-X)
Supplement: Supplementary appendix [file mmc1.pdf]

THE LANCET
Planetary Health

Supplementary appendix

This appendix formed part of the original submission and has been peer reviewed. We post it as supplied by the authors.

Supplement to: Aragón de León E, Shriwise A, Tomson G, et al. Beyond building back better: imagining a future for human and planetary health. *Lancet Planet Health* 2021; **5:** e827–39.

Supplementary Material

Search strategy and selection criteria

To empirically ground this Viewpoint, we conducted a rapid narrative review of English language literature published between December 2019 and October 2020. The review was guided by the following research question: “What is the impact of the COVID-19 pandemic on the Sustainable Development Goals (SDGs)?”, and it focused on literature published in the areas of social, economic, environmental, political and public health science. We used a semi-structured approach to scope the available evidence. The sources used to search for relevant literature included, but were not limited to: peer reviewed articles from LitCovid, by the U.S. National Center for Biotechnology Information (Pubmed), normative and policy guidance as well as country reports from the WHO database of publications on COVID-19, relevant reports and documents from databases of other United Nations (UN) agencies, intergovernmental and non-governmental organizations, websites from civil society organizations and media outlets, and other sources of grey literature. These sources were used to search for terms related to the research question, with a focus on the following: COVID-19, poverty, hunger and nutrition, health, education, gender, water and sanitation, energy, work and economy, industry, innovation and infrastructure, inequality, cities and communities, consumption, climate, biodiversity, peace, justice and institutions, and partnerships. Literature, reports and other material identified were included if they discussed the effect of the COVID-19 crisis with respect to one or more SDGs, with a particular focus on public health, social, economic, environmental, and political determinants of health.

The main findings from the rapid review were then grouped according to each SDG, and each SDG was then thematically categorized under the social (SDGs 1-5), economic (SDGs 7-12), and environmental (SDGs 6, 13-15) domains of sustainable development and under institutions (SDG 16) and partnerships (SDG 17) (Table 1). While there are many approaches to categorizing the

SDGs according to the three domains of sustainable development, we arrived at this classification by taking a mid-range approach between Folke et al.[1] and Morton et al.[2] Folke et al.[1] grouped the SDGs according to the categories of economy, society and the biosphere, while Morton et al.[2] drew from the 5Ps of sustainable development (people, prosperity, planet, peace, and partnership). To arrive at our classification, any SDGs that were consistently classified under society/people, economy/prosperity, and biosphere/planet in each of these respective approaches were categorized under the social, economic, and environmental domains, respectively. This left only three discrepancies. First, Folke et al.[1] classified SDG 6 (Clean water and sanitation) under 'biosphere', while Morton et al.[2] classified it under 'people'. In this case, we deferred to Folke et al.'s[1] classification under biosphere, as we considered water and sanitation as an environmental determinant of health, and therefore categorized it under the environmental domain. Second, Folke et al.[1] categorized SDG 7 (Affordable and clean energy) and SDG 11 (Sustainable cities and communities) under 'society', while Morton et al.[2] categorized it under 'prosperity'. In this case, we deferred to Morton et al.'s[2] classification, considering access to fuel and healthy settings, including housing in cities and communities, as critical determinants of health and well-being influenced by socio-economic factors. Therefore, SDGs 7 and 11 were categorized under the economic domain. Finally, while Folke et al.[1] included SDG 16 (Peace, justice and strong institutions) under 'society', we deferred to Morton et al.[2] and grouped SDG 16 and 17 together, as we view institutions and partnerships as analytically distinct from the more functional policy areas covered by the other SDGs. We fully appreciate that all of these categorizations are artificial, and in this case, we are using the domains of sustainable development as a heuristic device precisely to illuminate the nature of the interlinkages across these domains and to argue for a nested model configuration, rather than to create new siloes or argue for one form of classification over the other.

Additionally, to explore in greater depth how the disruption caused by COVID-19 presents opportunities for alignment towards a nested model, we selected three functional policy areas – food systems, transport and mobility, and work and incomes – as 'real time' emergent exemplars to illustrate key features of the E4As approach. We selected these due to both the extensive and widespread disruption they experienced early on in the pandemic through the containment measures and their potential impacts across all three domains of sustainable development.[3-5] As a result of both the extent of the disruption and highly political (as opposed to more regular and technical) nature of policy decision making in these areas, we viewed them as having significant transformative potential. We fully appreciate that these are not the only areas with such potential, and they are meant first and foremost to serve as illustrative examples in this Viewpoint. Furthermore, we do not wish to make any claims about whether or not transformative changes have indeed occurred in these areas.

Finally, building on the findings of Table 1 and Box 1, we also identified three systemic and transformative multipliers or accelerators: well-being economic models, social movements, and digital technological innovations. These were selected because of their potential to increase progress towards a nested model of sustainable development and to catalyze change across functional policy areas, including across the three illustrative functional policy areas mentioned above (i.e., food systems, transport and mobility, and work and incomes). We recognize that calls for transformative change that improves human and planetary health through well-being economic models, social movements and harnessing the potential of digital technologies are not new. Indeed, it is because these pre-existing accelerators had achieved a degree of momentum prior to the onset of COVID-19 that they have been further spotlighted during pandemic, for example, in calls for a green and inclusive economic recovery[6], the rapid roll-out and uptake of digital technologies[7], and the heightened role of social movements in re-shaping places and the distribution of power[8,9].

Moreover, we wished to explore these accelerators in greater depth and to highlight the need for continued scrutiny of the way they are employed throughout recovery precisely because changes in these areas have the potential, but are not pre-destined, to deliver a healthier future for people and the planet.

Table 1. Rapid narrative review of COVID-19 impacts on health and sustainable development

Impacts in the Social Domain	SDG
• Increased poverty and extreme poverty for the first time after 20 years of decline, resulting in households having fewer or no resources available for healthcare, healthy diets, and maintaining good housing conditions.[10,11]<br>• Relative poverty rates for informal workers predicted to rise from 34% before the crisis to 80% four months into the crisis.[12]<br>• Increased demand for and expansion of social protection including for informal workers.[10] As of 12 May 2020, 50 countries in Europe and Central Asia had utilized over 915 new or existing social protection programs to mitigate the consequences of COVID-19.[13] These measures included: advancing cash transfer payments, often on a one-off basis (e.g., Armenia and Turkey); expanding coverage of existing pension and other social protection schemes (e.g., Ukraine) and public works programs (e.g., Uzbekistan); increasing social insurance sickness benefits and extending health insurance coverage on a temporary basis (e.g., Kazakhstan) to guarantee access to healthcare services; subsidizing social protection contributions and unemployment insurance (e.g., Austria); and providing social benefits for employees, self-employed and domestic workers who have children aged up to 12 years and therefore had to be absent from work to care for them (e.g., Portugal).[13]	1 NO POVERTY
• Increased food insecurity. Sufficient access to diverse and nutritious food (e.g., fruits, vegetables, meat and dairy products) may be threatened, disproportionately affecting poor and nutritionally vulnerable groups as a result of upsurges in food prices and supply chains disruptions, as well as financial hardship.[10,14-17]	2 ZERO HUNGER
• Increased excess mortality from COVID-19 disease and other conditions due to the disruption in services and the inability of health systems to cope with increased demand, as well as the disruption in other sectors that determine health.[10,18-20]<br>• Based on a survey done in May–July 2020 by the WHO, on average countries of the WHO European Region reported at least partial disruptions in 49% of the 25 tracer health services. 89% of countries reported at least one disruption of essential service. Overall, the mean number of services disrupted per country was 11 out of 25.[21] Health systems overloaded by COVID-19 have also faced persistent organizational and procurement challenges.[10,21] Disruptions reported are due to a mix of supply and demand factors, such as limited access to public transportation. Concerns on the implications of delayed care and potential future outbreaks of infectious diseases among children as a result of disruptions in vaccination programs in some countries have been voiced by several stakeholders.[22,23]<br>• Increased risk of dying from COVID-19 for patients living with one or more chronic disease, including obesity, cardiovascular disease, diabetes or respiratory problems – diseases that are preventable with a healthy lifestyle.[24]<br>• Worsened mental health outcomes due to new stress factors and social isolation.[10,25-27]<br>• Reduced action and limited investment in public health priority areas other than COVID-19.[19]<br>• Health care workers are at higher risk of infection and of substantial increases in workload. Countries of the WHO European Region have used different strategies to mobilize additional staff and provide additional financial support and compensation to overloaded staff.[28-30]	3 GOOD HEALTH AND WELL-BEING
• Children have been excluded from schools in most European education systems with adverse consequences beyond interrupted learning,[31] including the loss of school meals and other school-based welfare measures. This affects children and families unequally, exacerbating the problems of those already experiencing vulnerability.[10]<br>• Exclusion from school may have long-term consequences for the learning, earning potential and well-being of individuals as well as for the health, economic, and social development of communities.[10]<br>• Potential negative developmental effects in children, adolescents and adults due to extensive isolation periods without peer contact and other social interaction.[10]	4 QUALITY EDUCATION
• While mortality rates for COVID-19 have been higher for men than for women, women are more likely to bear the brunt of the social and economic consequences of the pandemic.[32]<br>• Women are over-represented in informal work and in the most affected frontline sectors (e.g., health and social care, education, retail), increasing their vulnerability to health and economic hardships.[10,13]<br>• Increased harm from gender-based and domestic violence due to increased exposure to perpetrators and disruption to services to support survivors.[10,33]	5 GENDER EQUALITY

Impacts in the Economic Domain	SDG
• Increased volatility in global energy markets, with a major drop in demand for energy early on during the crisis due to disruption in different sectors of the economy, particularly transport, commercial and industrial operations. This decline is mostly attributed to reductions in economic activity rather than structural changes. Demand is projected to pick up as economic activity resumes – the full extent of the rebound is unknown and is projected to have substantial consequences for the energy transition.[34]	7 AFFORDABLE AND CLEAN ENERGY
• The COVID-19 pandemic is expected to have a more negative impact on the economy than the 2008 global financial crisis, and there are high levels of uncertainty regarding recovery.[35]<br>• Increase in wealth inequality driven by unprecedented falls in employment and total hours worked, with unequal impact among and within countries, communities, families, and individuals. As a result of the shutdowns, the International Labour Organization estimates for Europe and Central Asia a 17·5% loss in working hours for the second quarter of 2020, equivalent to 55 million full-time jobs.[36]<br>• In first month of the crisis, on average, informal workers worldwide lost as much as 60% of their earnings.[37]	8 DECENT WORK AND ECONOMIC GROWTH
• Accelerated investment in research and development for COVID-19 immunization and treatments and interruption and/or reduction of investment in research in other fields.<br>• Increased uptake of digital technologies, which have been a powerful enabler for business continuity in sectors like work, education, health, retail and increases in the online delivery of legal, social and civil services.[38] This has also laid bare the digital divide; for example, in Europe and Central Asia, at least 25 million schoolchildren have been unable to access remote learning.[39]	9 INDUSTRY, INNOVATION AND INFRASTRUCTURE
• Exacerbated inequalities leading to worsening health inequities, with a disproportionate impact on poorer and marginalized segments of the population, such as people with underlying health conditions, the elderly, homeless, people living in overcrowded residential settings, sex workers, people living in slums, unemployed people, migrants, and refugees.[40]<br>• Decrease in remittances in Europe and Central Asia in 2020 due to the combined effect of the pandemic and lower oil prices.[41]<br>• Low-paid migrant workers living in poor and crowded conditions experienced acute insecurities and account for a high proportion of COVID-19 infections in some countries.[42]	10 REDUCED INEQUALITIES
• As of July 2020, over 90% of COVID-19 cases were in urban areas.[37]<br>• Given the densely populated nature and complex systems of urban areas, restrictions in the use of public spaces resulted in a higher degree of disruption for people living in cities and other urban settlements as well as job losses and rearrangements, especially in the food and hospitality sectors.[37]<br>• Those who live in low-income neighborhoods and slums are at higher risk of exposure to infection from work in on-site essential jobs and are also more likely to live in multigenerational households with greater risk for intra-household transmission, as well as being disproportionately affected by the economic shocks.[37]<br>• Restrictions have resulted in record temporary reductions in noise, road accidents and air pollution. European data for some cities has shown a reduction of around 50%, and in some cases up to 70%, in NO_2 levels compared to pre-lockdown values.[43]<br>• Reduced air pollution between 21 February and 17 May is estimated to have averted 2 190 (1 960–2 420) premature deaths in Europe.[44] A positive number of long-term premature fatalities were and may continue to be avoided as a result of reduced $PM_{2.5}$ concentrations, with estimates ranging from 13 600 (11 900–15 300) to 29 500 (25 800–33 300), depending on the future scenarios of economic recovery adopted.[44]	11 SUSTAINABLE CITIES AND COMMUNITIES
• Patterns of consumption have changed, which has been forced or triggered by restrictions, reduced consumer confidence, unemployment and other macroeconomic effects. Data from the European Union shows some sections of the population are increasing their savings to account for uncertainty in income and as a result of reduced spending opportunities during COVID-19.[45] Reports from the private sector in Germany also indicate less use of shared-economy goods and more in-home consumption as a result of safety concerns.[46] The same study in Germany found differences according to household income and age group, with households with lower income not showing major changes in consumption habits and younger groups less interested in keeping the changes in consumption behaviour in the long-term.[46] The initial drop in consumer demand was shifted to higher levels of spending and consumption in some sectors, and there was a greater polarization of consumer spending.<br>• An increase in use of plastic disposables, both in general and due to medical waste, has been reported in some countries and cities.[47] Worldwide, the World Bank Group estimates the volume of medical waste has increased by up to 40%.[48] Overload in waste management systems has been reported in some countries in southeast Europe. Increases in medical waste can have serious health, safety, and environmental impacts, especially in countries where waste is burned or discarded in unregulated dumps.	12 RESPONSIBLE CONSUMPTION AND PRODUCTION

• On the other hand, industrial and commercial waste production has temporarily fallen drastically due to the slowdown in manufacturing activity.[48] Decline in demand of fresh water from large industrial and commercial users has also declined due to lockdowns and travel restrictions.[49]	

**Impacts in the Environmental Domain**	**SDG**
• Heightened awareness of both the extent and consequences of gaps in access, reliability, and the quality of water, sanitation, and hygiene, particularly in middle- and low-income countries.[50]<br>• With a few exceptions, investments in the water sector are projected to slow down worldwide as a result of shifts in demand patterns, supply disruptions, and various emergency measures employed by governments to cope with the pandemic.[49]	6 CLEAN WATER AND SANITATION
• Temporary reductions in carbon emissions and fossil fuels consumption were observed. The UN estimates a 6% reduction in greenhouse gas emission for 2020 – still below the 7·6% reduction needed to limit global warming.[51] While these numbers give a sense of progress, previous studies have shown that public support for environmental policies tends to be lower during economic recessions,[52] and the experience from previous global economic crises shows that such crises are typically followed by a large jump in emissions.[34]	13 CLIMATE ACTION
• Temporary reduction in water way traffic and fishery.[51]<br>• Increased plastic pollution in rivers and oceans as a result of increased use in disposables, such as personal protective equipment (e.g., disposable masks and gloves) and packaging for online purchases and take-away food.[53]	14 LIFE BELOW WATER
• While there has been a temporary reappearance of wild life in human settlement surroundings, the COVID-19 pandemic has highlighted the need to protect biodiversity, end deforestation, regulate animal trade and protect conservation areas and endangered species.[51]	15 LIFE ON LAND

**Impacts on Institutions and Partnerships**	**SDG**
• Extensive lockdowns that have been adopted to slow transmission of the virus restrict, by necessity, freedom of movement and, in the process, freedom to enjoy many other human rights. Such measures can inadvertently affect people's livelihoods and security, their access to health care (not only for issues related to COVID-19), food, water and sanitation, work, education, and leisure.[54]<br>• The crisis could result in the introduction of restrictions beyond those needed for the pandemic, and there is a need for transparency and evidence-informed decision making on the imposition and removal of restrictions. Rising ethno-nationalism, populism, authoritarianism and pushback against human rights have also been observed in some countries. [54] The instability and fear that the pandemic engenders is exacerbating existing human rights concerns, such as discrimination against certain groups, hate speech, xenophobia, attacks on and forced returns of refugees and asylum-seekers, mistreatment of migrants, sexual and gender-based violence, as well as limiting access to sexual and reproductive health services and rights.<br>• The implications of COVID-19 may further threaten global peace and security by exacerbating persistent political, social, and economic inequities that render some groups more vulnerable than others. Containment measures themselves have had a disproportionate impact on the poorest populations who often cannot work from home and were already living at subsistence levels. [40]	16 PEACE, JUSTICE AND STRONG INSTITUTIONS
• Global foreign direct investment is expected to have declined by up to 40% in 2020.[51] Adding to this scenario, as highlighted by the UN, most developing countries do not have sufficient domestic resources and fiscal space to adequately fund COVID-19 response and recovery measures. "No-one is safe until everybody is safe" means that international cooperation and external finance are crucial to enabling an equitable recovery.<br>• A number of international financial mechanisms have been rolled out by international financial institutions,[55] the UN[56] and philanthropies, among others, to support the COVID-19 response. However, the support has been insufficient to meet increased needs throughout COVID-19 response and recovery. Such mechanisms should operate in line with the Addis Ababa Action Agenda.[57]	17 PARTNERSHIPS FOR THE GOALS

Box 1: Illustrative examples of functional policy areas that have been disrupted by COVID-19, with scope for alignment during recovery and transition

Food systems

The COVID-19 pandemic has laid bare the vulnerabilities in our food system, leading to urgent calls to transform and reboot how we produce, process, transport, market and consume food and dispose of waste. Globally, food systems remain a driver of climate change; they are responsible for around 30% of greenhouse gas emissions and have contributed to substantial biodiversity loss.[58]

Some of the bottlenecks experienced during the crisis were due to sudden demand surges rather than a lack of capacity in the food supply.[59,60] At the same time, the need to ensure food security resulted in:

- Increased public attention on the precarious working conditions of low-wage food workers, many of whom are migrants or immigrants;[60]
- New and more direct ways of sourcing food and consequent growth of household and community food initiatives during lockdown, resulting in renewed interest in re-localization of food supply chains;[59]
- Actions by public authorities to secure food production and provision;[60]
- An upsurge of solidarity and community activism, with farmers' groups, and cooperatives organizing to buffer against crisis impacts, underlining the importance of engaging them in tackling climate change and other challenges;[60] and
- Unilateral import and export controls, highlighting the need to track such policies and assess their incidence, effectiveness and potential international spill over effects. In OECD countries, such measures are being evaluated with the Agricultural Market Information System to ensure transparency and timely information sharing on food trade and related supply chains.[61,62]

What started as crisis response must be transposed into food systems governance. To ensure transformative change in our food system, investments focused on resilience, rather than subsidies, are needed,[58] as well as fiscal incentives and public policies to attract private sector investment and to "re-localize" food supply chains. Agroecology and smart agricultural investments have been highlighted as systemic multipliers.[63] Current agricultural subsidies can be redirected into research investments in agroecology and into reducing inequalities in access to land.

The transformation needs to be guided by data and advanced analytics to ensure that investments are well-targeted in addition to providing access to reliable information to assess risks and allow for effective responses by policy makers, consumers, producers and traders. Digital services need to reach all farmers, including poor farmers, to ensure equitable access to knowledge, markets and financial support.

At the global level, the upcoming Food Systems Summit in 2021 is an opportunity for Member States to focus on how their national food systems can align with the 2030 Agenda and support SDG attainment. It will highlight the need to redesign global food systems, including a focus on food production, food loss and waste, and on negotiating dietary changes to improve human and planetary health.[64]

Transport and mobility systems

Whilst impacting negatively on travel-dependent businesses and industries,[65,66] restrictions in mobility have illuminated opportunities to change transport systems through demonstrated improvements in human and planetary health,[67] such as:

- Reduction in carbon emissions and air pollution around the world;[68]
- Reduction in work-related travel in light of the confirmed potential of virtual technology to support remote working and engagement; and
- Increases, in some instances, in cycling and walking as preferred modes of mobility.[69]

Transformations in transport and mobility systems go hand in hand with transformations in urban design and with co-benefits for business, productivity, the environment and health. Compact cities, with overlapping multifunctional layers, reduces travel demand. Evidence indicates higher retail footfall in areas with improved cycling infrastructure, as well as reductions in absenteeism and improved health from more active lifestyles.[70] Furthermore, investments in cycling and active mobility infrastructure have shown to be smart, sustainable and labour intensive, with a higher employment effect than that of general transport infrastructure.[70] Hepburn et al.[63], have highlighted the transformative potential of such policies for human and planetary health given their demonstrated ability to deliver on economic, social and environmental goals.

Work and incomes

COVID-19 has impacted work in multiple ways by changing patterns and modes of working, increasing unemployment (SDG 8)[71] and job insecurity, reducing working hours[36] and exacerbating pre-existing exclusion and inequalities (SDG 10).[72,73] The crisis has hit all sectors of society, but the young and lowest-paid have been hardest hit, and ongoing disruptions to education are likely to result in long-lasting impacts. Loss of income has consequences for housing security and access to healthcare, among other effects, leading to worse health conditions. Highlighting opportunities for transformative change for human and planetary health, the crisis has also:

- Revealed the importance of mitigation measures that enhance social protection, thereby tackling inequalities and poverty;[11]
- Expanded and increased utilization of social protection (e.g., social assistance, social insurance and labour market measures), including direct transfer programmes to impoverished and vulnerable households, in-kind transfers, robust social pension schemes, wage subsidies, regulations adjustments in labour market regulations, among others;[74]
- Simplified administrative systems to reduce barriers and enable universal access to social protection;[74] and
- Increased the value assigned to previously 'devalued' jobs, particularly in health, social care, retail and other jobs now deemed essential, some of which are known to have unsafe and unhealthy working conditions and to be done held predominantly by women.

Beyond the need to provide adequate social protection and the right to social security, recovery can also support job creation. While the fast-tracked adoption of digital technologies, and in the future, the ambition to transition to zero-carbon growth, can leave millions unemployed, countries can prepare and mitigate by designing recovery stimulus packages that create incentives for labor-intensive green businesses, industries and infrastructure projects, as well as through focusing on education and training to build human capital and resilience. Energy transition, sustainable transport, urban healthy settings, land restoration, forests and landscapes, health research and development are all labor-intensive investments that support a transformation towards a nested model of sustainable development.[63,75]

To ensure alignment towards improved human and planetary health, the use of short-term work/compensation/subsidies as a way to keep businesses afloat and tax relief measures can be further continued, with a focus on re-orienting the economy towards green jobs/industries. For example, tax incentives and disincentives can help tackle high polluters and compensation and subsidies should be conditioned on compliance with environmental and social benefits. Additionally, harnessing reshaped perceptions about work,[72] more flexible working arrangements and a consequent reduction in the need for mobility[76] can improve work-life balance and promote and enable the adoption of healthier life-styles. Alignment of investment in job creation and labour market policy should also be gender-sensitive. Based on current labour market structures, jobs in zero carbon infrastructure construction, for example, would most likely be male-dominated. To leave no one behind, educational gaps for students from disadvantaged backgrounds should also be addressed.[77]

References

1. Folke C, Biggs R, Norström AV, et al. Social-ecological resilience and biosphere-based sustainability science. *Ecol Soc* 2016;21(3):41. http://dx.doi.org/10.5751/ES-08748-210341.
2. Morton S, Pencheon D, Squires N. Sustainable Development Goals (SDGs), and their implementation: a national global framework for health, development, and equity needs a systems approach at every level, *Br Med Bull* 2017;124:81-90. https://doi.org/10.1093/bmb/ldx031.
3. Hale T, Angrist N, Goldszmidt R, et al. A global panel database of pandemic policies (Oxford COVID-19 Government Response Tracker). *Nat Hum Behav*, 2021;5:529-38. https://doi.org/10.1038/s41562-021-01079-8.
4. Rockström J, Sukhdev P. How food connects all the SDGs. https://www.stockholmresilience.org/research/research-news/2016-06-14-how-food-connects-all-the-sdgs.html. Date: 14 June 2016. Date accessed: 15 June 2021.
5. United Nations Economic Commission for Europe. *Recommendations for Green and Healthy Sustainable Transport – "Building Forward Better"*. Geneva: United Nations; 2021. https://unece.org/sites/default/files/2021-05/2101940_E_PDF_WEB.pdf. Date accessed: 15 June 2021.
6. Organisation for Economic Co-operation and Development. Focus on green recovery. http://www.oecd.org/coronavirus/en/themes/green-recovery. Date accessed: 6 December 2020.
7. Ting DSW, Carin L, Dzau V, et al. Digital technology and COVID-19. *Nat Med* 2020;26:459–61. https://doi.org/10.1038/s41591-020-0824-5.

8. Demaria F, Schneider F, Sekulova F, et al. What Is Degrowth? From an Activist Slogan to a Social Movement. *Environ Values*, 2013;22(2):191–215. www.jstor.org/stable/23460978.

9. World Health Organization. WHO Manifesto for a Healthy Recovery from COVID-19: Prescriptions and Actionables for a Healthy and Green Recovery. https://www.who.int/docs/default-source/climate-change/who-manifesto-for-a-healthy-and-green-post-covid-recovery.pdf. Date: May 2020. Date: 12 December 2020.

10. United Nations Committee for the Coordination of Statistical Activities. How COVID-19 is changing the world: a statistical perspective. Volume II. https://www.un.org/development/desa/pd/sites/www.un.org.development.desa.pd/files/unpd_2020_covid19-report-ccsa_vol2.pdf. Date: 1 September 2020. Date accessed: 17 December 2020.

11. Sumner A, Hoy C, Ortiz-Juarez E. Estimates of the impact of COVID-19 on global poverty. UNU-WIDER. WIDER Working Paper 2020/43. https://doi.org/10.35188/UNU-WIDER/2020/800-9. Date: April 2020. Date accessed: 17 December 2020.

12. International Labour Organization. ILO Monitor: COVID-19 and the world of work. Third edition. https://www.ilo.org/wcmsp5/groups/public/---dgreports/---dcomm/documents/briefingnote/wcms_743146.pdf. Date: 29 April 2020. Date accessed: 17 December 2020.

13. United Nations Regional Coordination Mechanism – Europe and Central Asia, United Nations Sustainable Development Group – Europe and Central Asia. COVID-19 and social protection in Europe and Central Asia: a moment of opportunity to expand and strengthen social protection mechanisms to safeguard health, well-being and livelihoods, leaving no

one behind. https://www.social-protection.org/gimi/RessourcePDF.action?id=56790. Date: 16 July 2020. Date accessed: 6 December 2020.

14. Moseley WG. The Geography of COVID-19 and a Vulnerable Global Food System. https://www.worldpoliticsreview.com/articles/28754/the-geography-of-covid-19-and-a-vulnerable-global-food-system. Date: 12 May 2020. Date accessed: 17 December 2020.
15. Headey D, Ruel M. The COVID-19 nutrition crisis: What to expect and how to protect. International Food Policy Research Institute Blog. https://www.ifpri.org/blog/covid-19-nutrition-crisis-what-expect-and-how-protect. Date: 23 April 2020. Date accessed: 17 December 2020.
16. Food and Agriculture Organization of the United Nations. Food system policy priorities and programmatic actions for healthy diets in the context of COVID-19. http://www.fao.org/3/ca9512en/CA9512EN.pdf. Date: 28 May 2020. Date accessed: 17 December 2020.
17. Headey D, Heidkamp R, Osendarp S, et al. Impacts of COVID-19 on childhood malnutrition and nutrition-related mortality. *Lancet* 2020;396(10250):519–21. https://doi.org/10.1016/S0140-6736(20)31647-0.
18. Lai AG, Pasea L, Banerjee A, et al. Estimated impact of the COVID-19 pandemic on cancer services and excess 1-year mortality in people with cancer and multimorbidity: near real-time data on cancer care, cancer deaths and a population-based cohort study. *BMJ Open* 2020;10(11):e043828. https://doi.org/10.1136/bmjopen-2020-043828.
19. Roberton T, Carter ED, Chou VB, et al. Early estimates of the indirect effects of the COVID-19 pandemic on maternal and child mortality in low-income and middle-income

countries: a modelling study. *Lancet Glob Heal* 2020;8(7):E901–8. https://doi.org/10.1016/S2214-109X(20)30229-1.

20. Giattino C, Ritchie H, Roser M, et al. Excess mortality during the Coronavirus pandemic (COVID-19). Our World in Data. https://ourworldindata.org/excess-mortality-covid. Date: 11 December 2020. Date accessed: 17 December 2020.
21. World Health Organization. Pulse survey on continuity of essential health services during the COVID-19 pandemic. https://apps.who.int/iris/rest/bitstreams/1297631/retrieve. Date: 27 August 2020. Date accessed: 17 December 2020.
22. COVID-19 Health Systems Response Monitor. How are countries reorganizing non-COVID-19 health care service delivery? https://analysis.covid19healthsystem.org/index.php/2020/05/06/how-are-countries-reorganizing-non-covid-19-health-care-service-delivery/. Date: 6 May 2020. Date accessed: 17 December 2020.
23. World Health Organization. Special feature: immunization and COVID-19. http://www.who.int/immunization/monitoring_surveillance/immunization-and-covid-19/en/. Date: June 2020. Date accessed: 17 December 2020.
24. Clark A, Jit M, Warren-Gash C, et al. Global, regional, and national estimates of the population at increased risk of severe COVID-19 due to underlying health conditions in 2020: a modelling study. *Lancet Glob Heal* 2020;8(8):E1003–17. https://doi.org/10.1016/S2214-109X(20)30264-3.
25. Nature Medicine. Keep mental health in mind. *Nat Med* 2020;26:631. https://doi.org/10.1038/s41591-020-0914-4.

26. Van Hoof E. Lockdown is the world's biggest psychological experiment – and we will pay the price. https://www.weforum.org/agenda/2020/04/this-is-the-psychological-side-of-the-covid-19-pandemic-that-were-ignoring/. Date: 9 April 2020. Date accessed: 17 December 2020.

27. Pan K-Y, Kok AAL, Eikelenbook M. et al. The mental health impact of the COVID-19 pandemic on people with and without depressive, anxiety, or obsessive-compulsive disorders: a longitudinal study of three Dutch case-control cohorts. *Lancet Psych* 2020; Online First. https://doi.org/10.1016/S2215-0366(20)30491-0.

28. Maier CB, Scarpetti G, Williams GA. What strategies are countries using to expand health workforce surge capacity to treat COVID-19 patients? Cross-Country Analysis - COVID-19 Health System Response Monitor. https://analysis.covid19healthsystem.org/index.php/2020/04/23/what-strategies-are-countries-using-to-expand-health-workforce-surge-capacity-to-treat-covid-19-patients/. Date: 23 April 2020. Date accessed: 15 June 2021.

29. Williams GA, Scarpetti G, Maier, CB. How are countries supporting the mental health and wellbeing of their health workforce? Cross-Country Analysis - COVID-19 Health System Response Monitor. https://analysis.covid19healthsystem.org/index.php/2020/05/21/how-are-countries-supporting-the-mental-health-and-wellbeing-of-their-health-workforce/. Date: 21 May 2020. Date accessed: 15 June 2021.

30. Organisation for Economic Co-operation and Development, European Commission. *Health at a Glance: Europe 2020. State of Health in the EU Cycle.* Paris: OECD Publishing, Paris; 2020. https://doi.org/10.1787/82129230-en.

31. United Nations Educational Scientific and Cultural Organization. Adverse consequences of school closures. https://en.unesco.org/covid19/educationresponse/consequences. Date accessed: 17 December 2020.
32. Burki T. The indirect impact of COVID-19 on women. *Lancet Infect Dis* 2020;20(8):P904–5. https://doi.org/10.1016/S1473-3099(20)30568-5.
33. World Health Organization. COVID-19 and violence against women: what the health sector/system can do. https://apps.who.int/iris/bitstream/handle/10665/331699/WHO-SRH-20.04-eng.pdf. Date: 7 April 2020. Date accessed: 17 December 2020.
34. International Energy Agency. *Sustainable Recovery. World Energy Outlook Special Report.* Paris: International Energy Agency. https://iea.blob.core.windows.net/assets/c3de5e13-26e8-4e52-8a67-b97aba17f0a2/Sustainable_Recovery.pdf. Date: June 2020. Date accessed: 15 June 2021.
35. International Monetary Fund. World Economic Outlook. A Crisis Like No Other, An Uncertain recovery. https://www.imf.org/-/media/Files/Publications/WEO/2020/Update/June/English/WEOENG202006.ashx. Date: June 2020. Date accessed: 17 December 2020.
36. International Labour Organization. ILO Monitor: COVID-19 and the world of work. Sixth edition. Updated estimates and analysis. https://www.ilo.org/wcmsp5/groups/public/@dgreports/@dcomm/documents/briefingnote/wcms_755910.pdf. Date: 23 September 2020. Date accessed: 17 December 2020.
37. United Nations. Policy Brief: COVID-19 in an Urban World. https://www.un.org/sites/un2.un.org/files/sg_policy_brief_covid_urban_world_july_2020.pdf. Date: July 2020. Date accessed: 17 December 2020.

38. United Nations Development Programme in Europe and Central Asia. COVID response: Digital transformation. https://www.eurasia.undp.org/content/rbec/en/home/coronavirus/regional-response-to-COVID19/digitaltransformation.html. Date: 2020. Date accessed: 17 December 2020.
39. United Nations Children's Fund. COVID-19: Are children able to continue learning during school closures? https://data.unicef.org/wp-content/uploads/2020/11/RemoteLearningFactsheet_Updated.pdf. Date: August 2020. Date accessed: 17 December 2020.
40. United Nations Department of Economic and Social Affairs. Responses to the COVID-19 catastrophe could turn the tide on inequality. https://www.un.org/development/desa/dpad/wp-content/uploads/sites/45/publication/PB-65.pdf. Date: May 2020. Date accessed: 17 December 2020.
41. World Bank Group and Knomad. COVID-19 Crisis Through a Migration Lens. Migration and Development Brief 32. http://documents1.worldbank.org/curated/en/989721587512418006/pdf/COVID-19-Crisis-Through-a-Migration-Lens.pdf. Date: April 2020. Date: 17 December 2020.
42. International Labour Organization. Social protection for migrant workers: A necessary response to the COVID-19 crisis. https://www.ilo.org/wcmsp5/groups/public/---ed_protect/---soc_sec/documents/publication/wcms_748979.pdf. Date: 23 June 2020. Date accessed: 17 December 2020.
43. World Health Organization. A new international day to celebrate clean air – and a sustainable recovery from COVID-19. https://www.euro.who.int/en/health-topics/environment-and-health/air-quality/news/news/2020/9/a-new-international-day-to-

celebrate-clean-air-and-a-sustainable-recovery-from-covid-19. Date: 7 September 2020. Date accessed: 17 December 2020.

44. Giani P, Castruccio S, Anav A, et al. Short-term and long-term health impacts of air pollution reductions from COVID-19 lockdowns in China and Europe: a modelling study. *Lancet Planet Heal* 2020;4(10):E474–82. https://doi.org/10.1016/S2542-5196(20)30224-2.

45. European Data Portal. Shedding Light on Changing Consumer Behaviour with Economic Data. https://www.europeandataportal.eu/en/impact-studies/covid-19/shedding-light-changing-consumer-behaviour-economic-data. Date: 19 May 2020. Date accessed: 17 December 2020.

46. Monitor Deloitte. Impact of the COVID-19 crisis on short-and medium-term consumer behavior. Will the COVID-19 crisis have a lasting effect on consumption? https://www2.deloitte.com/content/dam/Deloitte/de/Documents/consumer-business/Impact of the COVID-19 crisis on consumer behavior.pdf. Date: June 2020. Date accessed: 17 December 2020.

47. United National Conference on Trade and Development. Growing plastic pollution in wake of COVID-19: how trade policy can help. https://unctad.org/news/growing-plastic-pollution-wake-covid-19-how-trade-policy-can-help. Date: 27 July 2020. Date accessed: 17 December 2020.

48. International Finance Corporation. COVID-19's Impact on the Waste Sector. https://www.ifc.org/wps/wcm/connect/dfbceda0-847d-4c16-9772-15c6afdc8d85/202006-COVID-19-impact-on-waste-sector.pdf?MOD=AJPERES&CVID=na-eKpI. Date: June 2020. Date accessed: 17 December 2020.

49. International Finance Corporation. The Impact of COVID-19 on the Water and Sanitation Sector. https://www.ifc.org/wps/wcm/connect/126b1a18-23d9-46f3-beb7-047c20885bf6/The+Impact+of+COVID_Water%26Sanitation_final_web.pdf?MOD=AJPERES&CVID=ncaG-hA. Date: June 2020. Date accessed: 17 December 2020.

50. United Nations Economic Commission for Europe. COVID-19: the role of the Water Convention and the Protocol on Water and Health. https://www.unece.org/environmental-policy/conventions/water/envwater/covid-19-the-role-of-the-water-convention-and-the-protocol-on-water-and-health. Date: 3 December 2020. Date accessed: 17 December 2020.

51. United Nations. The Sustainable Development Goals Report 2020. https://unstats.un.org/sdgs/report/2020/The-Sustainable-Development-Goals-Report-2020.pdf. Date: 2020. Date accessed: 17 December 2020.

52. Benegal S, Scruggs L. Economic Conditions and Public Opinion on Climate Change. *Oxf Res Encycl of Climate Sci*. https://doi.org/10.1093/acrefore/9780190228620.013.310. Date: 22 November 2016. Date accessed: 17 December 2020.

53. Hughes K. Protector or polluter? The impact of COVID-19 on the movement to end plastic waste. https://www.weforum.org/agenda/2020/05/plastic-pollution-waste-pandemic-covid19-coronavirus-recycling-sustainability/. Date: 6 May 2020. Date accessed: 17 December 2020.

54. United Nations. COVID-19 and Human Rights: We are all in this together. https://unsdg.un.org/sites/default/files/2020-04/COVID-19-and-Human-Rights.pdf. Date: April 2020. Date accessed: 17 December 2020.

55. Segal S, Negus O. Tracking International Financial Institutions' Covid-19 Response. https://www.csis.org/analysis/tracking-international-financial-institutions-covid-19-response. Date: 21 July 2020. Date accessed: 17 December 2020.

56. United Nations. UN COVID-19 Response & Recovery Fund. http://mptf.undp.org/factsheet/fund/cov00#. Date: 17 December 2020. Date accessed: 17 December 2020.

57. United Nations. Addis Ababa Action Agenda of the Third International Conference on Financing for Development. https://sustainabledevelopment.un.org/content/documents/2051AAAA_Outcome.pdf. Date: 27 July 2015. Date accessed: 17 December 2020.

58. United Nations. Policy Brief: The Impact of COVID-19 on Food Security and Nutrition. https://www.un.org/sites/un2.un.org/files/sg_policy_brief_on_covid_impact_on_food_security.pdf. Date: June 2020. Date accessed: 17 December 2020.

59. Petetin L. The COVID-19 Crisis: An Opportunity to Integrate Food Democracy into Post-Pandemic Food Systems. *Eur J Risk Regul* 2020;11(Special Issue 2):326–36. https://doi.org/10.1017/err.2020.40.

60. International Panel of Experts on Sustainable Food Systems. COVID-19 and the crisis in food systems: Symptoms, causes, and potential solutions. http://www.ipes-food.org/_img/upload/files/COVID-19_CommuniqueEN%282%29.pdf. Date: April 2020. Date accessed: 17 December 2020.

61. Organisation for Economic Co-operation and Development. COVID-19 and international trade: Issues and actions. https://read.oecd-ilibrary.org/view/?ref=128_128542-

3ijg8kfswh&title=COVID-19-and-international-trade-issues-and-actions. Date: 12 June 2020. Date accessed: 17 December 2020.

62. Organisation for Economic Co-operation and Development. Transparency and policy co-ordination: Valuable lessons on averting a food crisis. http://www.oecd.org/coronavirus/en/data-insights/transparency-and-policy-co-ordination-valuable-lessons-on-averting-a-food-crisis. Date: 16 October 2020. Date accessed: 17 December 2020.
63. Hepburn C, O'Callaghan B, Stern N, et al. Will COVID-19 fiscal recovery packages accelerate or retard progress on climate change? *Oxf Rev Econ Pol* 2020;36(Issue Supplement 1):S359–81. https://doi.org/10.1093/oxrep/graa015.
64. Gralak S, Spajic L, Blom I, et al. COVID-19 and the future of food systems at the UNFCCC. *Lancet Planet Health* 2020;4(8):E309–11. https://doi.org/10.1016/S2542-5196(20)30163-7.
65. International Labour Organization. COVID-19 and road transport. ILO Sectoral Brief. https://www.ilo.org/wcmsp5/groups/public/---ed_dialogue/---sector/documents/briefingnote/wcms_746914.pdf. Date: June 2020. Date accessed: 17 December 2020.
66. International Civil Aviation Organization. Effects of Novel Coronavirus (COVID-19) on Civil Aviation: Economic Impact. https://www.icao.int/sustainability/Documents/COVID-19/ICAO_Coronavirus_Econ_Impact.pdf. Date: 17 December 2020. Date accessed: 17 December 2020.
67. United Nations Economic Commission for Europe. Mobility Management: A guide of international good practices. https://thepep.unece.org/sites/default/files/2020-

04/Mobility%20Management_WEB.pdf. Date: March 2020. Date accessed: 17 December 2020.

68. Chen K, Wang M, Huang C, et al. Air pollution reduction and mortality benefit during the COVID-19 outbreak in China. *Lancet Planet Heal* 2020;4(6):E210–2. https://doi.org/10.1016/S2542-5196(20)30107-8.

69. Von Hakan Volkan E, Blackburn A. Data Sources on Coronavirus impact on transport https://wiki.unece.org/display/DSOCIOT/Data+Sources+on+Coronavirus+impact+on+transport. Date: 16 November 2020. Date accessed: 17 December 2020.

70. Portugal-Pereira JO, Doll CNH, Suwa A, et al. The sustainability mobility – Congestion nexus: a co-benefits approach to finding win-win solutions. Transport and Communications Bulletin for Asia and the Pacific 2013;82:19–31. https://www.unescap.org/sites/default/files/bulletin82_Article-2.pdf.

71. Organisation for Economic Co-operation and Development. Unemployment rate forecast. https://data.oecd.org/unemp/unemployment-rate-forecast.htm. Date accessed: 17 December 2020.

72. Kramer A, Kramer KZ. The potential impact of the Covid-19 pandemic on occupational status, work from home, and occupational mobility. *J Voc Behav* 2020;119:103442. https://doi.org/10.1016/j.jvb.2020.103442.

73. United Nations Department of Economic and Social Affairs. World Economic Situation And Prospects: June 2020 Briefing, No. 138, https://www.un.org/development/desa/dpad/publication/world-economic-situation-and-prospects-june-2020-briefing-no-138/. Date: 1 June 2020. Date accessed: 17 December 2020.

74. Gentilini U, Almenfi M, Orton I, et al. Social Protection and Jobs Responses to COVID-19: A Real-Time Review of Country Measures. World Bank. https://openknowledge.worldbank.org/handle/10986/33635. Date: 2020. Date accessed: 17 December 2020.
75. Bhattacharya A. From rescue to recovery, to transformation and growth: building a better world after COVID-19. https://www.lse.ac.uk/GranthamInstitute/news/from-rescue-to-recovery-to-transformation-and-growth-building-a-better-world-after-covid-19/. Date: 27 April 2020. Date accessed: 17 December 2020.
76. Paul K. Twitter announces employees will be allowed to work from home 'forever.' https://www.theguardian.com/technology/2020/may/12/twitter-coronavirus-covid19-work-from-home. Date: 12 May 2020. Date accessed: 17 December 2020.
77. Stern N, Unsworth S, Valero A, et al. Strategy, investment and policy for a strong and sustainable recovery: An action plan. https://www.lse.ac.uk/granthaminstitute/wp-content/uploads/2020/07/Strategy-investment-and-policy-for-a-strong-and-sustainable-recovery.pdf. Date: 2 July 2020. Date accessed: 17 December 2020.
